# Thrombopoietin Secretion by Human Ovarian Cancer Cells

**DOI:** 10.1155/2017/1873834

**Published:** 2017-03-30

**Authors:** Samaher Besbes, Shahid Shah, Iman Al-dybiat, Shahsoltan Mirshahi, Helene Helfer, Haythem Najah, Caroline Fourgeaud, Marc Pocard, Ibtissem Ghedira, Jeannette Soria, Massoud Mirshahi

**Affiliations:** ^1^Lariboisière Hospital, University of Sorbonne Paris Cité-Paris Diderot 7, INSERM U965, 75010 Paris, France; ^2^Faculty of Pharmacy, University of Monastir, 5000 Monastir, Tunisia; ^3^Diagnostica Stago, 92230 Gennevilliers, France

## Abstract

The thrombopoietin (TPO) gene expression in human ovary and cancer cells from patients with ovarian carcinomatosis, as well as several cancer cell lines including MDA-MB231 (breast cancer), K562 and HL60 (Leukemic cells), OVCAR-3NIH and SKOV-3 (ovarian cancer), was performed using RT PCR, real-time PCR, and gene sequencing. Human liver tissues are used as controls. The presence of TPO in the cells and its regulation by activated protein C were explored by flow cytometry. TPO content of cell extract as well as plasma of a patient with ovarian cancer was evaluated by ELISA. The functionality of TPO was performed in coculture on the basis of the viability of a TPO-dependent cell line (Ba/F3), MTT assay, and Annexin-V labeling. As in liver, ovarian tissues and all cancer cells lines except the MDA-MB231 express the three TPO-1 (full length TPO), TPO-2 (12 bp deletion), and TPO-3 (116 pb deletion) variants. Primary ovarian cancer cells as well as cancer cell lines produce TPO. The thrombopoietin production by OVCAR-3 increased when cells are stimulated by aPC. OVCAR-3 cell's supernatant can replace exogenous TPO and inhibited TPO-dependent cell line (Ba/F3) apoptosis. The thrombopoietin produced by tumor may have a direct effect on thrombocytosis/thrombosis occurrence in patients with ovarian cancer.

## 1. Introduction

Thrombosis is a major complication in malignant diseases [1, 2]. More than 50 years ago, Levin and Conley reported that thrombocytosis was associated with breast, lung, digestive, and ovarian cancers [3]. Hemostatic disorders in cancer result from the capacity of tumor cells to secrete procoagulant factors and to interact with blood components such as platelets [4, 5]. While platelets are essential for normal hemostasis, their unbridled activation may result in thrombus formation leading to thrombocytosis complications. Moreover, patients with elevated platelet count have a higher risk to develop venous thromboembolism [6].

Beside their role in coagulation, platelets are also involved in cancer growth and dissemination at different levels [7]. Thus, activated platelets are considered as an important source of lysophosphatidic acid (LPA), which has been shown to be involved in promotion of bone metastasis in a model of mouse bearing breast or ovarian cancer cells [8].

Thrombopoietin (TPO) is a key regulator of megakaryopoiesis and megakaryocyte progenitor proliferation by promoting stem cell differentiation into megakaryocytes and their expansion, hence, boosting platelet production [9, 10].

TPO is mainly produced by the liver and it is also secreted by kidney, bone marrow, and spleen [11]. The human TPO gene is localized on chromosome 3q27 and comprises six exons and five introns [12–14]. To date, 8 different variants of TPO mRNA have been identified, including the full length mRNA (TPO-1) and its 7 alternative splicing variants. Proliferative activity was highlighted only in TPO-1 isoform [15].

Moreover, TPO seems to be more than a megakaryopoiesis regulator. Indeed, TPO has been admitted as a crucial regulator of proliferation and secretory activity in porcine ovarian follicular cells [16]. In pathologic conditions, several cancer cell lines from lung, stomach, liver, and thyroid human carcinomas express the TPO gene [17]. In a case report, Furuhashi et al. reported that TPO could be produced by ovarian carcinoma [18]. Tsukishiro and his colleagues observed, in a comparative study, that plasma TPO concentration may be a biomarker that distinguishes between benign tumor patients and those with malignant ovarian cancer [19]. It was already described that TPO level increased by an inflammatory process mediated by IL-6, produced by macrophages and monocytes, dose-dependently increases TPO mRNA levels in hepatoma cell lines [20]. However, the presence of thrombopioetin detected by immunohistochemistry is attributed to the capture of thrombopoietin due to thrombopoietin receptor expression in human cancer cells [21].

Previously, we detected TPO release in an adenocarcinoma cell line culture medium (NIH:OVCAR-3 cell line: abbreviated OVCAR-3 in this study). We also observed that activated protein C (aPC), a natural anticoagulant, increased OVCAR-3 TPO secretion [22].

The main goal of this study was to analyze TPO gene expression in ovarian cancer and to assess whether the ovarian TPO produced by cancer cells is functional or not.

## 2. Materials and Methods

### 2.1. Cell Culture

#### 2.1.1. Cell Lines

The human cancer cell lines used were ovarian (OVCAR-3 a poorly differentiated serous carcinoma cell line and SKOV-3 an endometrioid cancer cell line), breast (MDA-MB231 and MCF7), gastric (AGS, KATO-III), intestinal (LS174T), lung (A549), leukemic (myeloid leukemia K567 and promyelocytic leukemia HL60), and cervical (HELA). We also used human microvascular endothelial (HMEC-1) and interleukin-3- (IL-3-) dependent murine (Ba/F3) cell lines. Cell lines were obtained from American Type Culture Collection (ATCC, Manassas, VA). The Ba/F3 cell line that expresses the human thrombopoietin receptor (MPL) was kindly provided by Caroline Marty and Isabelle Plo [23]. Cells were cultured in RPMI 1640 medium containing 10% heat-inactivated fetal bovine serum (FBS), 50 ug/ml of streptomycin, 50 IU/ml of penicillin, and 2 nM of L-glutamine (Gibco, Saint Aubin, France). Cells were incubated at 37°C in a humidified atmosphere containing 5% CO_2_.

#### 2.1.2. Conditioned Media

Cells were seeded in plates or flasks, grown to 80% confluency, and then incubated in serum-free culture medium. Three culture conditions were assayed: (1) in presence of protein C (PC) (Protexel, Courtaboeuf, France) at a concentration of 10 *µ*g/ml, (2) in presence of activated protein C (aPC) (Xigris, Suresnes, France) also at a concentration of 10 *µ*g/ml, and (3) without any addition as control. Cell flasks were incubated 5 hours prior to flow cytometry analysis and cells in plates were incubated 24 hours for coculture experiments. Ba/F3 cell line was cultured in the presence of (1) recombinant IL-3 (5 ng/ml) (Sigma-Aldrich, Saint-Quentin Fallavier, France), (2) human recombinant thrombopoietin (TPO) (50 ng/ml) (LifeSpan Biosciences, USA), (3) PC (10 *µ*g/ml), and (4) aPC (10 *µ*g/ml).

#### 2.1.3. Evaluation of TPO by ELISA

After culture, the cells were carefully washed with phosphate-buffered saline (PBS) and then cells were cultured without fetal calf serum or additional growth factor in culture flask. After 18 hours, the cells were collected and soluble extracts were tested for TPO determination. The TPO was quantified using the commercially available ELISA (R & D Systems Quantikine Human TPO ELISA kit, Abingdon, UK), according to the manufacturer's instructions for cell culture supernatants. The results were expressed in pg/ml/1 × 10^6^ cells.

#### 2.1.4. Coculture of Cancer Cells and Ba/F3 Cells

OVCAR-3, MDA-MB23,1 and K562 were cultured in bottom two cell culture compartments separated by a 0.4 *µ*m micropore membrane (Fisher Scientific, Illkirch, France) and Ba/F3 cells were cultured in the upper compartment.

#### 2.1.5. Ascites Cell Culture of Patients

Ascitic fluids from patients were provided by the digestive surgery department of Lariboisiere Hospital (Paris, France). All patients gave their written informed consent. Clinical and biological annotations were recorded in an Access database approved by the “*Commission Nationale de l'informatique et des Libertés, *France.” A total of 6 cancer patients were included in the study. Medical records reported that 4 patients had cancer from ovarian origin. Only one patient had a signet ring cell gastric carcinoma. All patients were admitted for surgery during a period from October 2014 to February 2015. Each ascitic sample was centrifuged and the cell pellet obtained was cultured in flasks coated with 0.2% gelatin (Sigma, France) in DMEM medium (GIBCO, Saint Aubin, France), supplemented with 20% of heat-inactivated fetal bovine serum (FBS), 50 ug/ml of streptomycin, 50 IU/ml of penicillin, and 2 nM of L-glutamine.

### 2.2. Gene Expression

#### 2.2.1. Primers Selection, PCR, and Nested-PCR

**Figure 1:**
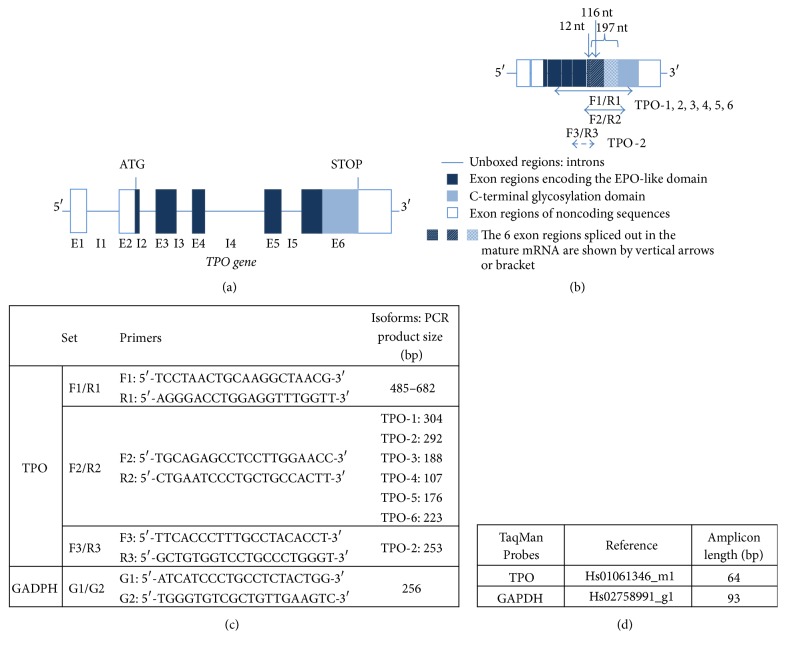
TPO primers' selection. (a, b) Schematic illustration of human TPO gene and mRNA isoforms and selected TPO primers. (a) TPO gene contains 6 exons (E 1–6) and 5 introns (I 1–5). (b) Alternative RNA splicing patterns previously identified for TPO. Horizontal arrows represent the amplified regions by RT-PCR. (c) Primers used for PCR and nested-PCR. First PCR products were used as DNA template for the nested-PCR. TPO-amplified isoforms and their sizes are shown. GADPH PCR was used as control. (d) TaqMan Probes for TPO and GADPH.

TPO primers, for PCR and nested-PCR, were selected from Sasaki et al. study [17]. Our choice was based on TPO gene structure and its possible splice variants as shown in Figures 1(a) and 1(b). Specific primers for TPO were synthetized by Eurofins Genomics (Ebersberg, Germany). Primers used for PCR and nested-PCR are shown in Figure 1(c). The detection of TPO gene expression was investigated by using F1/R1 primers set which amplifies in a common product (all TPO splice variants). We performed also PCR for Glyceraldehyde 3-phosphate dehydrogenase (GAPDH) to ensure integrity and quantitative comparison of cDNAs. GAPDH cDNA was amplified using specific primers (G1/G2) from Eurofins Genomics (Figure 1(c)). TPO gene expression was investigated by the detection of TPO mRNA in cells from patient's ascites culture or in cell lines. The cell RNA extracts were prepared using the RNeasy kit (Qiagen, Courtabœuf, France). Ovarian total* RNA* and liver* cDNA* from nontumor human adult tissues were provided by BioChain (Newark, USA). Following reverse transcription (Mu-MLV reverse transcriptase and oligo (dT) primers), the polymerase chain reaction (PCR) was performed with MasterMix (5 Prime, Düsseldorf, Germany). The PCR products (using F1/R1 primers, Figure 1), along with a 100 bp DNA ladder, were analyzed by electrophoresis on agarose gels containing GelRed nucleic acid gel stain. After electrophoretic separation of the products, we selectively excised only intense bands for nested-PCR. DNA was eluted and purified using a DNA gel extraction kit (Norgen Biotek Corp, Ontario, Canada). Nested-PCR was performed using F2/R2 or F3/R3 primers and the products were analyzed by agarose gel electrophoresis. The DNA bands of interest were again selected and excised and DNA was purified. Purity and concentration of RNA or DNA samples were determined by optical density measurement and the ratio of 260 : 280 nm using the NanoDrop™ 2000c spectrophotometer (Thermo Fisher Scientific, France).

#### 2.2.2. Sequencing of Nested-PCR Products

Nested-PCR extracted DNA products were sequenced by Eurofins Genomic (Ebersberg, Germany), using the cycle sequencing technology (dideoxy chain termination/cycle sequencing) on ABI 3730XL sequencing machine. Sequences were analyzed by the Basic Local Alignment Search Tool (BLAST) in the NCBI database.

#### 2.2.3. Real-Time PCR

TPO gene expression was also analyzed by real-time PCR and TaqMan® primers with FAM probes for TPO or GADPH (Figure 1(d)) were from Applied Biosystems (France). The real-time PCR was performed using TaqMan gene expression assay and LightCycler® 96 real-time PCR System (Roche Diagnostics GmbH, Mannheim, Germany). Analysis of gene expression was performed using the comparative C_T_ method [24].

### 2.3. Flow Cytometry

#### 2.3.1. Protein Quantification

OVCAR-3 and MDA-MB231 were cultured in flasks and incubated in a serum-free medium with or without PC/aPC stimulation as described above. Protein transport inhibitor, containing Brefeldin-A, provided by BD Biosciences (Le Pont de Claix, France), was added (1 *µ*l/ml) or not to the culture medium. Then, cells were detached with accutase, washed with phosphate-buffered saline (PBS), and suspended in 100% heat-inactivated FBS. Cell fixation and permeabilization procedures were performed using the Perfix-nc assay kit as indicated by the manufacturer (Beckman Coulter, France). Cells were mixed at first with an anti-hTPO primary antibody (1 : 200) (Sigma-Aldrich, Saint-Quentin Fallavier, France), then washed with PBS, and incubated with a fluorescein isothiocyanate (FITC) coupled secondary antibody (1 : 100). Cells were then washed twice and the pellet was resuspended in 500 *µ*l of buffer provided in the kit. Flow cytometry was done using a standard Canto II eight-color flow cytometer (Becton Dickinson Biosciences, France) at 530 nm and data analysis was performed using Flowjo software (Flowjo LLC, USA).

#### 2.3.2. Cell Viability

Ba/F3 cells were cocultured separately with MDA-MB231 or OVCAR-3 or K562 cells, in the presence or absence of 10 *μ*g/ml PC or aPC, as indicated above. Ba/F3 cells were cultured with IL-3, TPO, PC, or aPC and served as control. After 72 h, Ba/F3 cells were collected from each well and washed with PBS and then fixed with 4% (final concentration) formaldehyde. Then, Ba/F3 cells were suspended in cold binding buffer (10 mM HEPES pH 7.4, 140 mMNaCl, 2.5 mM CaCl2, 0.1% BSA), incubated for 15 min at 4°C with FITC conjugated Annexin-V (SouthernBiotech, Birmingham, USA), and shielded from light. Data acquisition and analysis were performed by flow cytometry as described above.

### 2.4. MTT Viability Test

Ba/F3 cells were cocultured with OVCAR-3 or MDA-MB231or K562 cells in the presence of PC or aPC. Ba/F3 cells were cultured with TPO and served as control. After 72 h, Ba/F3 cell viability was investigated using Thiazolyl Blue Tetrazolium Bromide colorimetric assay (MTT) according to Tada et al. protocol [25].

### 2.5. Statistical Analysis

All values reported are the average ± SEM. Statistical significance was determined using the GraphPad Prism 6.0 software (Kruskal-Wallis test/Student's *t*-test) and *P* < 0.05 was considered statistically significant.

## 3. Results

### 3.1. TPO Expression by Ovarian Carcinomatosis in Primary Culture and Cell Lines

**Figure 2:**
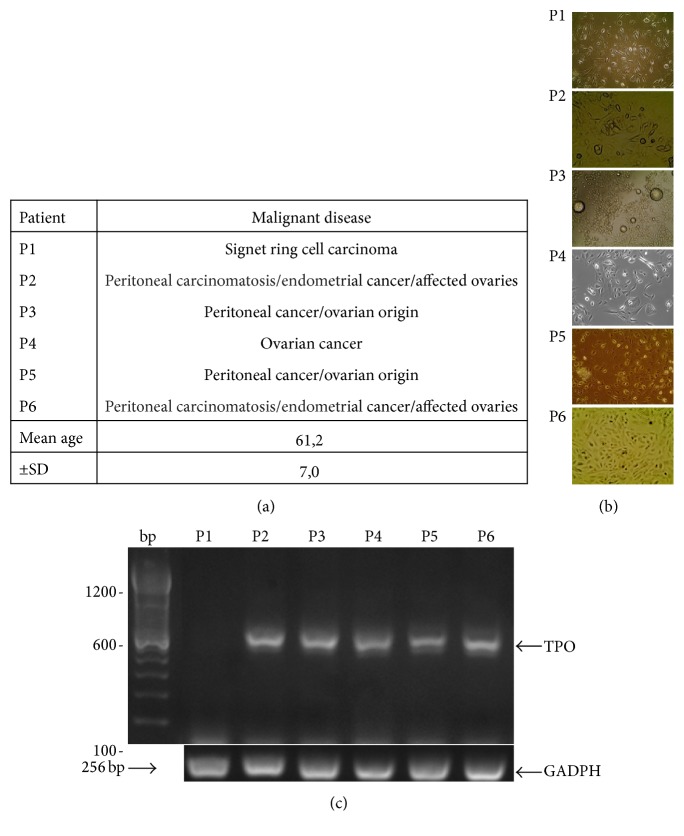
TPO gene expression in cultured cells from ascitic fluids of cancer patients. (a) Subject data. (b) Photographs taken of ascitic fluid cells in culture. (c) Analysis of TPO and GADPH gene expression. 2% agarose gel. PCR using F1/R1 primers for TPO gene amplification.

**Figure 3:**
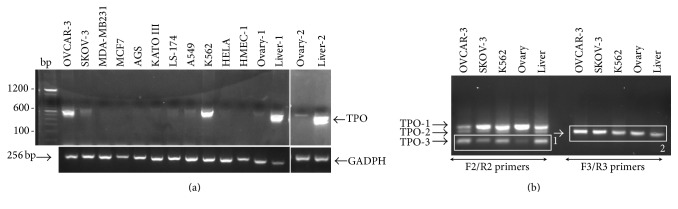
TPO gene expression by cell lines. (a) First PCR analysis of TPO and GADPH gene expressed by various cell lines, ovarian (OVCAR-3 and SKOV-3), breast (MDA-MB231 and MCF7), gastric (AGS, KATO-III), intestinal (LS174T), lung (A549), leukemia (K562), cervical (HELA), and human microvascular endothelial (HMEC-1) cell lines. 2% agarose gel. Normal adult ovary (1 and 2) and liver (1 and 2) tissues served as control. (b) 2% agarose gel pattern of nested-PCR product of TPO: TPO-1 (full length), TPO-2 (12 bp deletion), and TPO-3 (116 bp deletion). Boxes (1, 2) represent bands chosen for sequencing.

TPO gene expression was analyzed in cultured cells harvested from peritoneal fluids of six patients suffering from carcinomatosis. Clinical characteristics of patients are summarized in Figure 2(a). Photographs taken from each patient cultured cells are presented in Figure 2(b). RT-PCR analysis showed that, unlike the signet ring cells, all the cells from ovarian origin expressed the TPO gene (Figure 2(c)). To extend this analysis, we inspected TPO gene expression in various human cancer cell lines. Results showed different TPO expression with regard to the cell lines considered: ovarian (OVCAR-3 and SKOV-3) and leukemia (K562) cancer cell lines expressed TPO at high level; gastric (AGS, KATO-III), intestinal (LS174T), lung (A549), and cervical (HELA) cancer cell lines expressed TPO at a very low level; TPO gene expression could not be detected in human breast adenocarcinoma (MDA-MB231 and MCF7) and in human microvascular endothelial (HMEC-1) cell lines (Figure 3(a)). TPO gene expression was found to be lower in normal ovary tissue than in the liver.

### 3.2. Positive TPO Cell Lines Express Different Transcript Patterns and Contained TPO

**Figure 4:**
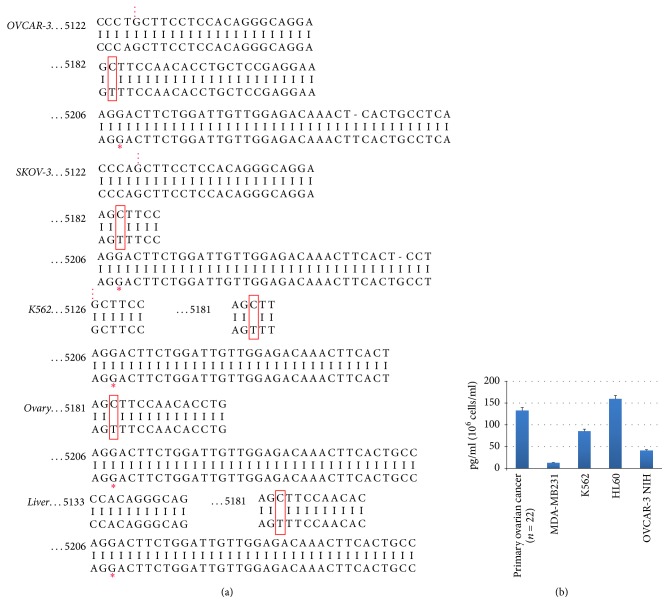
Comparison of TPO-3 sequence as well as TPO levels in the soluble cancer cells extracts. (a) Sequences of PCR products extracted from agarose gel bands are shown in the upper line. R2 primer was used for sequencing. Boxes show a C/T 5183 SNP. “…” and “*∗*” symbols refer to two different alternative splicing sites (12 bp and 116 bp deletions, resp.). A 116 bp deletion characterizing TPO-3 is detected. BLAST reveals no significant mutation. (b) TPO levels in the soluble cancer cells extracts evaluated by enzyme-linked immunosorbent assay (ELISA) for 10^6^ cell/ml (the mean of three experiments) from OVCAR-3 NIH cell line, breast cancer MDA-MB231 cell line (as control), myeloid leukemia K562, promyelocytic leukemia HL60 cell lines, and primary ovarian carcinomatosis (*n* = 22).

We next compared the different TPO splice variants in the cancer cell lines that were positive for elevated TPO expression (OVCAR-3, SKOV-3, and K562) and in control ovarian tissues. Results showed that all cancer cells lines express the three TPO-1 (full length TPO), TPO-2 (12 bp deletion), and TPO-3 (116 pb deletion) variants, similarly to the liver and ovary control tissues (Figure 3(b)). Since the splice variants differ by a limited number of nucleotide, the presence of these variants was confirmed by sequencing. Of note, sequencing data showed that the TPO-3 variant harbored the C/T 5183 SNP (Figure 4(a)) which is known to be a common mutation for all clones [14] that does not affect the final sequence of the protein.

We further quantified the amount of TPO synthetized by ovarian cancer cells OVCAR-3, by the cells from peritoneal carcinomatosis (*n* = 22), as well as leukemic cells K567 and HL60. The MDA cells were used as control. The results presented in Figure 4(b) show that TPO levels in the soluble extract of ovarian cancer are significantly higher than in control.

### 3.3. Regulation of TPO Production by Activated Protein C

**Figure 5:**
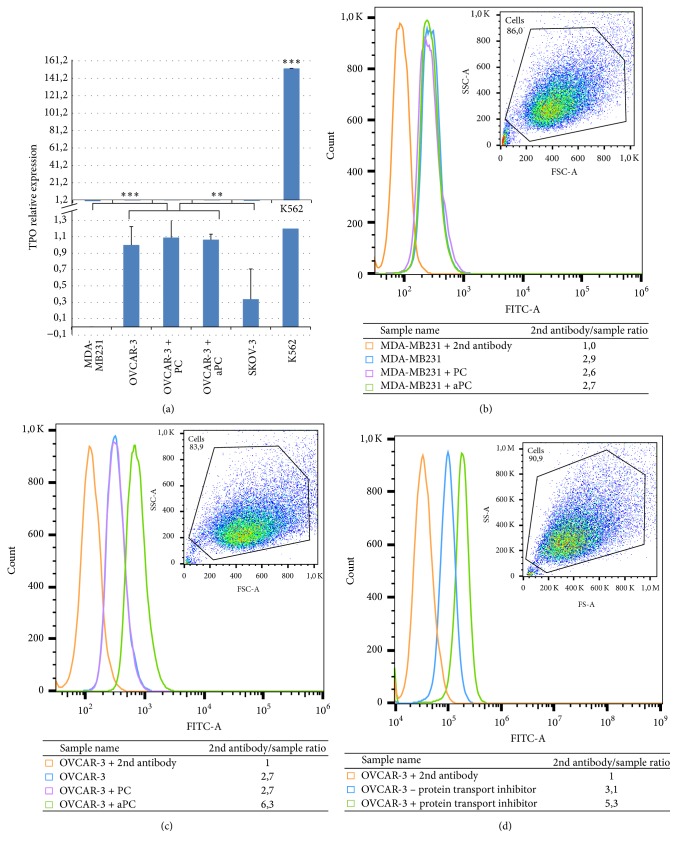
TPO gene expression in the presence or absence of protein C. (a) Quantification of TPO gene expression using TaqMan Probes in different cancer cell lines such as MDA-MB231 (as control), OVCAR-3, SKOV-3, and K562. Nonsignificant results were observed in the presence of protein C (*P* < 0,05). High TPO gene expression was observed in leukemic K562 cell. Kruskal-Wallis test (^*∗∗*^*P* < 0,02, ^*∗∗∗*^*P* < 0,01). Flow cytometry distribution plots are shown. Graphs represent Geometric Fluorescence Mean. (b, c): graphs for MDA-MB231 (b) and OVCAR-3 (c) cells incubated with the secondary antibody alone (orange), labeled with primary and secondary antibodies after incubation without PC or aPC (blue), and with PC (purple) or with aPC (green). (d) TPO-released protein by OVCAR-3. OVCAR-3 were incubated (for 5 hours) without (blue) or with (green) protein transport inhibitor or with only 2nd antibody as control (orange). 2nd antibody GMFI/GMFI sample ratio was calculated for each condition.

Since we previously showed that aPC-stimulated OVCAR-3 produced TPO by cytokine array [22], we further investigated the regulation of TPO expression by PC and aPC assessed by quantitative PCR in OVCAR-3 before and after stimulation by protein C (PC) or activated protein C (aPC). The results obtained showed that neither PC nor aPC had any effect on TPO mRNA level (Figure 5(a)). To further explore TPO production, we next analyzed TPO protein content in these various condition by flow cytometry. We considered MDA-MB231 cell line as a negative control (Figure 5(b)). We observed that TPO contents of OVCAR-3 cells were similar before and after PC stimulation. However, TPO secretion increased markedly when the cells were incubated with aPC (Figure 5(c)). The results comparing the TPO protein content between OVCAR-3 cells cultured with or without a protein transport inhibitor (Figure 5(d)) allow to conclude that TPO is secreted by the cells.

### 3.4. OVCAR-3 Secretes Functional TPO

To assess whether TPO from OVCAR-3 cells is functional, the TPO-dependent Ba/F3 cell line was cocultured with OVCAR-3, in the presence or absence of PC or aPC. Ba/F3 was cocultured with MDA-MB231 cells that do not produce TPO (as control) or with K562 that produce high level of TPO or cultured alone with PC, aPC, exogenous TPO, or interleukin-3 (IL-3). Ba/F3 cells viability was assessed in those various conditions by MTT assay and Annexin-V labeling. Viability of Ba/F3 cells in each condition was compared with that of Ba/F3 cells incubated with exogenous TPO. The results showed that the viability of Ba/F3 cells was identical when cocultured with OVCAR-3 cells with or without PC stimulation but increased when cocultured with OVCAR-3 stimulated by aPC.

**Figure 6:**
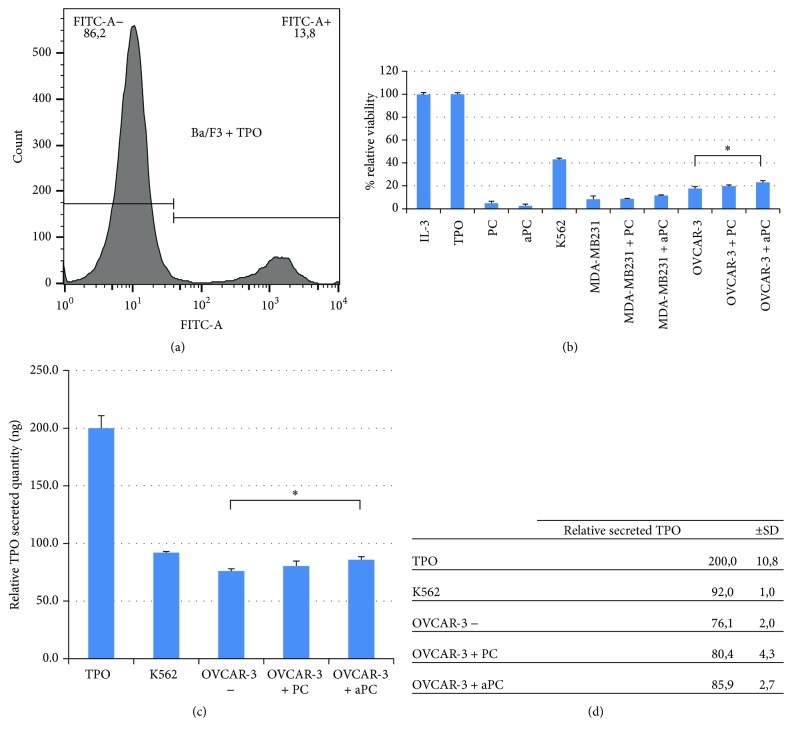
Functionality of TPO: viability study of TPO-dependent Ba/F3 cells. (a) The panel shows the distribution of two populations of Ba/F3 cells cultured in the presence of TPO: a nonlabeled population FITC-Annexin-V (viability) and a labeled population FITC-Annexin-V (apoptosis). (b) The graph shows Ba/F3 viability (not stained by FITC-Annexin-V). (c, d) Relative TPO secreted quantity (ng). (*P* < 0,02). Student's *t*-test (^*∗*^*P* < 0,05).

In addition Ba/F3 also survived when incubated with IL-3 (Figures 6(a) and 6(b)). Using MTT assay, we evaluated the amount of TPO produced by OVCAR-3 stimulated or not by aPC, by comparing the survival of Ba/F3 cells incubated with xenogeneic TPO and cocultured with aPC-stimulated OVCAR-3 (Figure 6(c)). Relative TPO secreted (ng) is indicated in Figure 6(d).

These observations indicated that the TPO secretion via aPC is mediated by guanine nucleotide exchange factor (GBFI).

## 4. Discussion

The results presented here confirm that normal ovarian tissue as well as ovarian cancer cells expresses TPO and show for the first time that TPO produced by cancer cells is functional. These results provide new insight into the relationship between cancer and hemostatic disorders.

Thrombocytosis associated with malignant disease was, traditionally, attributable to interleukin-6 (IL-6) or to granulocyte-macrophage colony-stimulating factor [26–28]. Stone et al. suggested that IL-6 may act alone in a paracrine manner to increase hepatic thrombopoietin production, thereby increasing platelet count [28].

Furthermore, Sakar et al. demonstrated the expression of TPO and its receptor c-MPL in bovine ovarian follicles. They also showed that TPO and c-MPL expression and production in the corpus luteum, during oestrous cycle, vary depending on the luteal stage [29]. Therefore, locally produced TPO and c-MPL may play a crucial role in the regulation of the platelets generation during oestrous cycle.

In addition, TPO serum levels are more elevated in women with ovarian cancer than those with benign ovarian cyst [19, 30]. Here we showed that normal ovarian tissue, ovarian cancer nodule, and the ovarian cancer cell lines, especially OVCAR-3, express TPO. These results are reminiscent from a preliminary observation done in ovarian carcinoma [18].

TPO gene expression in cancer cell lines from different origins also is not identical. Ovarian or leukemic cell line expressed more TPO. Curiously, as observed by RT-PCR and flow cytometry, the amount of the TPO extracted from ovarian cancer nodule is higher that its cell line OVCAR-3 or myeloid leukemia K567 and promyelocytic leukemiaHL60 cell line. In contrast no TPO was extracted from breast cancer cell line (MDA-MB231), compared with ovarian or leukemic cells. Previously we found that when OVCAR-3 cells were incubated with activated protein C, the cancer cell migration was upregulated via MEK-ERK and Rho-GTPase pathway signalization [31] and the amount of secreted TPO in the culture medium detected by cytokine array increased four times [22]. In nonpublished results, we also demonstrated that activated protein C and, to a lesser degree, protein C induced the microparticles release in OVCAR-3 cell line. Here again we observed an upregulation of TPO synthesis in the presence of activated protein C in culture medium. Interestingly, when the cancer cell is preincubated in the presence of aPC with Brefeldin-A, an inhibitor of protein transport from endoplasmic reticulum to golgi apparatus [32, 33], TPO secretion was inhibited. These observations indicated that the TPO secretion via aPC is mediated by guanine nucleotide exchange factor (GBFI).

In parallel studies, the level of the TPO in the plasma of patients with ovarian cancer (*n* = 25) was significantly increased, compared with normal plasma levels of TPO (results not shown).

The sequence analysis of TPO genetic materials in the cell lines confirms that the ovarian cancer cells lines as well as leukemic cells expressed the three TPO-1 (full length TPO), TPO-2 (12 bp deletion), and TPO-3 (116 pb deletion) variants. We do not observe any modification of gene sequences compared with the liver and ovary origin. A TPO-3 variant (C/T 5183 SNP) is known to be a common mutation.

Concerning functional activity analysis of TPO secreted by ovarian cancer cells, we showed that the coculture of ovarian cancer cell line in conditional medium with a TPO-dependent Ba/F3 cell line could decrease the Ba/F3 cell apoptosis due to secretion of TPO from OVCAR-3 cells.

In addition, we report for the first time that the pattern of expression of the TPO gene in ovarian cancer cells is similar to that observed in the liver, and most importantly that the TPO produced is functional.

These results have two major clinical implications.

- First, TPO could be used as a biomarker for the detection and progression of ovarian pathology. Indeed, data suggest hypothesis whereby TPO-secreting ovarian cancer cells contribute significantly to the elevation of TPO plasma level in patients with ovarian cancer. Further study should be performed to establish a quantitative relationship between TPO plasma level and cancer progression.
- Second, the production of functional TPO by ovarian cancer cells may be responsible for the risk of thromboembolism or thrombocytosis in patients with ovarian cancer. In such a context, it is most likely that the TPO produced by the cancer cells directly act to promote the expansion of platelets.
